# Origin, Definition and Division of Tissues

**Published:** 1882-06

**Authors:** C. Heitzman

**Affiliations:** New York.


### ARTICLE II.

### *Origin, Definition and Division of Tissues.*

BY C. HEITZMANN, OF NEW YORK.

The formation of tissues is a question much discussed by biologists and embryologists. New, and it is expected plain views can be obtained concerning this difficult matter, by reducing the germ of the higher animals into the schema of a single lump of bioplasson, f. i., an amœba. Such an atttempt of simplifying the ideas of the significance of the "germinal layers" is made in this article.

ORIGIN.

All complex organisms—the higher developed animals—originate from an ovum of the female impregnated by the admixture of spermatozoids of the male. The ovum being enclosed by a hyaline layer (zona pellucida of Von Baer,) is composed of living matter, in reticular arrangement (the germ of Remak,) which contains a nucleus-like body, the vesicula germinativa with a varying number of coarser granules, the nucleoli, the maculæ germinativæ. In mammals and some amphibia, the germ *in toto* is transformed into the animal, whereas in the eggs of birds, scaly amphibia and osseous fishes, a portion of the germ is changed into yolk which serves as a pabulum. After the spermatozoids have entered the germ and after fructification has taken place, its living matter increases rapidly, the vesicula germinativa disappears, and the germ, by a process of division, splits at first into two portions, seperated from each other by a light, narrow rim, but connected by extremely delicate filaments which traverse the light rim. Each half of the germ splits into a number of lumps which, in the same manner as the first half, remain connected; thus the segmentation of the ovum results in the formation of numerous corpuscles, which by collecting in a flat layer represent the germinal disk of Pander in the germ of the impregnated egg of the chicken. The segmentation was first observed by Prevost and Dumas (1824,) in the ovum of frog; by Coste (1848,) in the ovum of fowl, and by Bischoff (1842,) in the ovum of mammals. According to the last-named observer the subdivision into smaller elements in the rabbit's germ does not go on uniformly throughout its whole extent, inasmuch as in the germ a cavity is formed around which the elements of segmenta tion accumulate, in order to build up the germ-membrane proper with a slightly thickened spot, the germ hill of Von Baer.

The first differentiation of the germ-disk, or the germ-membrane, consists in the formation of layers, of which at first two, shortly afterward three, are recognizable. The formation of such layers became known first through the researches of Caspar Friedrich Wolff (1768,) who claimed that the whole system of the intestines is developed from simple laminæ. Pander, in 1817, perfected the theory of Wolff; he knew that after hatching had continued for twenty-four hours, three easily separable layers could be found in the germ-membrane. Von Baer, in 1822, described four layers, of which the two upper he termed the animal, the two lower the vegetative. Remak, in 1855, maintained that the germ-membrane of the impregnated but unhatched egg consists of two layers, and that, upon hatching, the lower is again split into two layers, the lower of which lines the one above it like an epitheial cover. Having ascertained the individuality of each of these three layers, he endeavored to find out their relation to the developing organs; he called the upper layer the horny or sensorial; the middle layer the motorial and germinative; the under layer intestinal and glandular. According to S. Stricker's researches (1860–1870,) the original under layer of Remak consists—at least above the germ-cavity, and before the middle layer has made its appearance—of only a single stratum of flattened cells, and the formation of the middle layer is due to the immigration between the two layers of new cells. He termed the upper layer of Remak the combined horny and nervous layer, as he found that in batrachia the horny layer is quite distinct from the nervous layer, the former being uniformly thin, the latter, on the contrary, thickened even in the earliest stages in that part where later the brain is formed. He is unable to confirm, despite of Remak's positive assertions, that nervous elements are also developed from the middle layer.

Stricker (Manual of Histology, American edition, 1872,) in speaking of the development of the fowl's germ, says: "The cells of the under layer change their form and arrangement during the first hours of incubation. They become flattened, and, when seen in tranverse section, appear spindle-shaped. Hence, after incubation has gone on for a few hours, we can ascertain beyond even the shadow of a doubt that there are two, and only two, layers. * * * The under layer, immediately after its separation from the subdivided germ, consisted in some places of a single thickness of cells, while in other places, in a transverse section, small heaps of cells could be recognized projecting from the layer. * * * Peremeschko, however, has made the communication that the large grandular cells, lying on the bottom of the germ-cavity, increase very considerably in numbers during the first hours of incubation. Now, since with this increase in numbers there is not at the same time a corresponding diminution in size, it is very natural to suppose that the cells which project from the under germ-layer fall to the bottom of the cavity. This suggestion appears all the more probable when we recall the fact that some of the elements of segmentation, which are situated in the lower portion of the germ, remain lying at the bottom of the cavity at the time when the germ, in the production of this very cavity, separates itself from the adjacent parts. * * * We are led to conjecture that the grandular bodies, which before lay at the bottom of the cavity, have found their way to the space between the two first germ-layers." Stricker, based upon Oellacher's researches, says that similar relations are also found in the trout's germ.

At present investigators agree that the body of vertebrates is at first a flat sheet consisting of three main layers, for the designation of which the following names have been proposed; *epiblast*, the upper layer; *mesoblast*, the middle layer, and *hypoblast* the under layer. Of these the epiblast and hypoblast are very thin, composed of but one layer of plastids, whereas the mesoblast is a bulky heap of plastids, all of which are interconnected and represent the main mass of the future organism. As the originally flat sheet of the germ becomes curved downwards so that the two lateral halves are bent toward the median line, where they grow together, cavities are formed in the interior of the germ, which are lined by the under layer and its derivatives. The horny layer furnishes the external covering of the body and lining of the external glands, while the under layer provides the lining of the intestinal cavity and its glandular organs. Linings of this description are called "epithelia," and it follows that the epiblast and the hypoblast give rise to all epithelial formations (including the crystalline lens;) the hypoblast to those of the intestines and their glandular elongation and accumulations. The main bulk of the body is a product of the mesoblast; from it proceed the tissues termed connective tissue, which exclusively contains blood and lymph vessels, muscles and nerves, the latter arising from the uppermost portions of the mesoblast.

DEFINITION.

In comparing the earliest formations of the germ with a single plastid, formerly called a "unicellular organism," or a "protoplasmic body," such as the amœba, valuable hints may be obtained as to the significance of the three germinal layers. The amœba is covered by an extremely thin layer of living matter. If the amœba be flattened out and bent, its cover will represent the upper and under thin layer of the germ, which exclusively serves as an investing layer of both the outer surface and all cavities of the body, being directly or indirectly connected with the outer world. The main bulk of the amœba is living matter in reticular arrangement with thickened points of intersection of the threads of the network; this matter, retaining in the mesoblast and its derivations its reticular shape, furnishes in higher organisms, as a result of a sort of division of labor, the tissues. The nature of the tissues is determined: firstly.

by the manner in which the living matter is distributed, and secondly, by the chemical changes of the fluid contained in the meshes of the reticulum. Tissues are complex formations of living matter in a network arrangement. The meshes of the network contain a liquid which allows the living matter to exhibit contractility in a high degree, as in muscles and nerves, or the network contains a more or less solidified basis substance, which limits its contractility as in the solidified basis substance, mainly serves as a support for the more active tissues (muscles and nerve), and as a carrier of liquids in closed spaces.

### DIVISION.

According to this view, there are but four elementary tissues in the animal body. All these are interconnected and built up on one and the same plan.

1. *Connective tissue.* In this the reticulum of living matter contains in its meshes a more or less solid, nitrogenous (glue-yielding) basis substance; while points of intersection rich in living matter, suspended in a liquid, represent the connective tissue corpuscles. Of all tissues, only the connective tisssue carries in closed vessels the liquids which serves for nutrition, such as blood and lymph. Aside from this and acting as support for other tissues, its physiological activity is relatively small.

2. *Muscle tissue.* The reticulum of living matter at its points of intersection consist of more or less regularly distributed large prismatic, cylindrical or granular thickenings (sarcous elements,) connected by thin filaments, while the meshes contain a liquid which admits of powerful contractions of the living matter in large territories. This tissue is the motor apparatus proper. It is accompanied by and attached to connective tissue, carrying the vessels.

3. *Nerve tissue.* Here the living matter is arranged in the shape of either a very delicate reticulum with very small points of intersection (ganglionic corpuscles, gray matter,) or in delicate solid cords (axis cylinders,) while the meshes contain a liquid which allows the living matter in limited territories to contract rapidly. This tissue serves as an apparatus of sense impression, intellect, and sensory and motor conduction. It is largely accompained by and mixed with connective tissue, carrying blood vessels.

4. *Epithelial tissue.* The reticulum of living matter is very delicate, and arranged in flat layers, which at certain regular intervals contain a horny cement-substance. The function of epithelial tissue is to cover the surface and the cavities of the body ; it alone serves as apparatus of secretion, and for the formation of the essential parts of reproduction : spermatozoids and ovum.—*New England Journal of Dentistry.*

